# Editorial: Food Oral Processing and Nutrition Through the Lifespan

**DOI:** 10.3389/fnut.2021.702724

**Published:** 2021-06-29

**Authors:** Elsa Lamy, Ana Carolina Mosca, Paula Midori Castelo

**Affiliations:** ^1^Mediterranean Institute for Agriculture, Environment and Development, University of Évora, Évora, Portugal; ^2^Department of Agricultural, Food, Environmental and Animal Sciences, University of Udine, Udine, Italy; ^3^Department of Pharmaceutical Sciences, Universidade Federal de São Paulo, São Paulo, Brazil

**Keywords:** food oral processing, mastication, nutrition, eating behavior, saliva

A balanced healthy diet is recognized as essential to prevent several non-communicable diseases, such as diabetes, hypertension, cardiovascular diseases or even some types of cancer. The need to promote shifts to healthier diets is even more relevant in the actual context, where obesity rates are increasing worldwide. Effective nutritional strategies, aimed at promoting healthier eating habits are warranted, from young to old ages. But for this purpose, a good comprehension of the factors involved in food preferences and choices is required.

Food oral processing comprises the sequence of transformations that food undergoes inside the mouth and will influence food sensory perception and food digestion. In oral processing, the food structure is first deformed and degraded by the forces applied by the teeth and soft tissues, including the tongue. Following, the fragments formed upon chewing are mixed with saliva, producing a bolus that can be safely swallowed. During this dynamic and synchronized process, the continuous interactions between oral structures, saliva and food produce the multiple sensations that are processed by humans into sensory perception. Moreover, the way the different food structures are broken and the food pieces are mixed with saliva affects (favoring or limiting) the biological availability and absorption of nutrients.

Masticatory performance influences the amount and type of food eaten, and, consequently, nutrition. An association between reduced chewing performance and obesity has been reported in the literature, with a higher consumption of soft high-fat and high-refined carbohydrates foods being associated to a reduced masticatory function. Masticatory function may vary according to several factors, among which oral health, age and sex. This can be particularly important in critical periods, such as the childhood, when food habits are defined, and at old age, where the compromised masticatory function will affect nutritional status. There are reports of better masticatory performance in normal-weight than in overweight children, with poor masticatory performance being also related with being underweight. For the elderly, masticatory difficulties resulting from teeth loss can lead to the risk of malnutrition. The design of new foods, able to overcome these problems, is a necessity to guarantee adequate nutrition.

Food oral processing has a major role in flavor perception and the participation of saliva in this process has been increasingly noticed in recent years. The role of this fluid in oral sensory perception started to be recognized in the case of astringency. Although the exact mechanism is still not completely known, this oral tactile sensation results from the interaction between salivary proteins with astringent molecules, disrupting the lubricating properties of saliva. Astringency usually results in food rejection. The way food is broken and mixed with saliva will affect the availability of astringent compounds to interact with salivary proteins and, consequently, affecting astringency intensity and food acceptance. Moreover, a relationship between salivary constituents and basic tastes has been observed, as well as an influence of this fluid in the way the volatile molecules are released during food breaking by mastication. This suggests that the way food is mixed with saliva will influence food acceptance and the consequent development of dietary habits.

The present Research Topic provides a collection of high-quality manuscripts presenting different aspects of food oral processing and its interaction with sensory perception, eating behavior and nutrition at different ages. This issue is composed of 9 manuscripts, including 7 original research articles, one review and one perspective article from Asia, Europe, and South America.

The article from Marquezin et al. presents evidence about how morbid obesity is related with oral health and nutritional patterns, with individuals with higher body mass indexes presenting poorer dietary habits and behaviors. Possebon et al. investigated the effect of implant-retained mandibular overdenture in oral parameters and quality of life, concluding that the impact of oral rehabilitation on masticatory function should consider the facial morphology.

The relationship between saliva and food oral perception is also present in this Research Topic. Schwartz et al. review compiled the different works that describe how diet influences saliva antioxidant capacity and how the latter influence food perception. Okawa et al. presented data suggesting that chewing strokes and saliva flow rate are associated with the concentration of aroma released through the retronasal route.

The importance of texture for food acceptance and nutritional intake can be found in two original articles, from Tournier et al. and Schwartz et al. The first work assessed how the type of food presentation (puree, small pieces, etc.) modulates further texture acceptance in 4–36-month-old children, while the study of Schwartz et al. was designed to investigate the role of food presentation (texture) in satiation and subsequent intake in schoolchildren.

In line with the need of new food products that guarantee nutritional quality through lifespan, three different articles focus on the importance of food oral processing to achieve acceptance. Amoah et al. compared breads of increasing nutrient quality and Tejada et al. evaluated the effect of reducing the amount of salt in the acceptance of Iberian chorizo. Both studies showed the possibility of changing products to healthier options. De Lavergne et al. gave the perspective from industry point of view of providing benefits for various target populations. The authors highlight how scientific understanding about food oral processing is important for product development, even more when specific population groups are considered. Besides presenting the different methodologies that are generally used for assessing food oral processing, this article focuses on the current tendency of an increasing demand for plant-based products, what illustrates the relevance of deeper, multidisciplinary and integrated knowledge about the processes occurring in the mouth.

Overall, the studies presented in this Research Topic focus on different aspects of food oral processing, considering different age-group populations, and showing the relevance of food transformation in the mouth in the context of a healthier nutrition.

## Author Contributions

EL, AM, and PC proposed the scope and concept of the present Research Topic, invited researches, contributors, handled the submissions, and approved the final version. PC was the main corresponding Editor. EL prepared the main draft of this Editorial, which was reviewed by AM and PC. All authors contributed to the article and approved the submitted version.

## Conflict of Interest

The authors declare that the research was conducted in the absence of any commercial or financial relationships that could be construed as a potential conflict of interest.

